# MapEff: An Effective Graph Isomorphism Agorithm Based on the Discrete-Time Quantum Walk

**DOI:** 10.3390/e21060569

**Published:** 2019-06-05

**Authors:** Kai Liu, Yi Zhang, Kai Lu, Xiaoping Wang, Xin Wang, Guojing Tian

**Affiliations:** 1 College of Computer, National University of Defense Technology, Changsha 410073, China; 2 Science and Technology on Parallel and Distributed Processing Laboratory, National University of Defense Technology, Changsha 410073, China; 3 College of Computer Science and Electronic Engineering, Hunan University, Changsha 410082, China; 4 Institute of Computing Technology, Chinese Academy of Sciences, Beijing 100190, China

**Keywords:** graph isomorphism, isomorphic mapping, discrete-time quantum walk, graph mining, data mining

## Abstract

Graph isomorphism is to determine whether two graphs have the same topological structure. It plays a significant role in areas of image matching, biochemistry, and information retrieval. Quantum walk, as a novel quantum computation model, has been employed to isomorphic mapping detection to optimize the time complexity compared with a classical computation model. However, these quantum-inspired algorithms do not perform well—and even cease to work—for graphs with inherent symmetry, such as regular graphs. By analyzing the impacts of graphs symmetry on isomorphism detection, we proposed an effective graph isomorphism algorithm (MapEff) based on the discrete-time quantum walk (DTQW) to improve the accuracy of isomorphic mapping detection, especially for regular graphs. With the help of auxiliary edges, this algorithm can distinguish the symmetric nodes efficiently and, thus, deduct the qualified isomorphic mapping by rounds of selections. The experiments tested on 1585 pairs of graphs demonstrated that our algorithm has a better performance compared with other state-of-the-art algorithms.

## 1. Introduction

Graph isomorphism is to figure out whether a pair of graphs has the same structure. It is known as an equivalence relationship on graphs, and, as such, it divides the class of all graphs into some equivalence classes. The set of graphs isomorphic to each other is described as an isomorphism class of graphs and has the same properties. Graph isomorphism has a pivotal role in various areas, including image matching [1], biochemistry [2], information retrieval [3], and other fields [4]. Up to now, whether this problem is one of nondeterministic polynomial (NP)-time-complete problems is still an open question [5]. This study of the graph isomorphism algorithm focuses on a major challenge faced by many researchers: how to reduce computational complexity.

Previous studies on graph isomorphism using classical computation model have not proposed a solution with the polynomial complexity for any kinds of graphs. Although the algorithms PlanarGI [6] and TreeGI [7] can deal with graph isomorphism in polynomial time, they just target planar graphs and tree graphs separately. For general graphs, there are some influential isomorphism algorithms, including VF2 [8], VF3 [9], LAD [10], RI [2], and Nauty algorithm [11]. The key point of their design is by trimming with backtracking and heuristics. By utilizing the procedure of verification in the exploring, they add verified unit bijections to the isomorphic mapping one after another, and eventually obtain a correct isomorphic mapping. Although these algorithms are perfectly accurate, their complexity is not so ideal and may become exponential in some special scenarios. In fact, most classical graph isomorphism algorithms have a high computational complexity. Therefore, researchers seek to explore an efficient graph isomorphism algorithm with polynomial time complexity.

Recent development of quantum computation has provided some new ideas for the algorithm design. As a novel quantum computation model, quantum walk has been applied to the graph isomorphism and make the polynomial complexity a reality. These algorithms have been proposed based on the simulation of discrete-time quantum walk (DTQW) or continuous-time quantum walk (CTQW), and have achieved a satisfying performance on the experimental accuracy. Most of these existing methods can be classified into two categories. One is to find all unit bijections by performing a round of quantum walk simulation on a big auxiliary graph, and the auxiliary graph is constructed through connecting all the vertex pairs with auxiliary nodes. For example, two methods [1,12] proposed by Emms utilized this strategy. The other is to find the isomorphic mapping through conducting quantum walk simulation multiple times on two graphs, and the algorithms proposed by Qiang [13] are the representative. In a different way, each quantum walk simulation can figure out a pair of matching nodes.

Although these quantum-inspired algorithms have the polynomial time complexity for all kinds of graphs, their accuracy on those graphs with symmetry is still one of the greatest challenges. For symmetric graphs, such as regular graphs, have many equivalent nodes with the same degree. Because quantum walks cannot distinguish the equivalent nodes very well, these algorithms could not exactly detect the isomorphic mapping function between the regular graphs. Therefore, the strongly similar graphs affect the accuracy of this kind of algorithm. In the last year, we proposed a novel algorithm named IsoMarking [14] by marking vertices to distinguish the symmetric nodes to reduce the harmful effect of symmetry in regular graphs. However, marking process for each node introduced a heavy complexity, thus a low efficiency, especially in large number of nodes. Besides, the limited marking types in IsoMarking cannot adequately reduce the symmetry of the regular graph and will restrict the performance of this algorithm. To address these problems, we proposed an effective graph isomorphic mapping algorithm (MapEff), inspired by Emms’ algorithm. MapEff utilized auxiliary edges to help detect isomorphism mapping, which not only eliminate the harmful impacts of symmetry but also can choose the correct unit bijections based on the verified matching nodes. After unit bijections are established one by one, the whole isomorphism mapping function is obtained. It is hoped that this study will contribute to the graph isomorphism, especially on regular graphs, and the main contributions of this paper are listed as follows:

1. We proposed the idea of adding auxiliary edges based on verified unit bijection to reduce the harmful impact of symmetric graph, thus the ability to distinguish equivalent nodes exactly.
2. We designed a practical mechanism to select the correct unit bijection based on the verified mapping nodes in isomorphic mapping.
3. In the experiments on 1585 pairs of graphs, we discovered that MapEff not only figure out the isomorphic mapping perfectly between ordinary graphs but also has a satisfactory accuracy on regular graphs.

The rest of this paper is organized as follows. Section 2 introduces some basic concepts and presents related isomorphism algorithms based on the quantum walk. Section 3 is concerned with the restriction of graph’s symmetry in isomorphism detection and then gives the detailed procedures of the MapEff algorithm, as well as a further discussion about this algorithm. In Section 4, we compare the accuracy of MapEff with other state-of-art isomorphism algorithms to show its advantages. Lastly, Section 5 concludes and proposes some further thoughts beyond the scope of this study.

## 2. Preliminary and Related Work

In this section, we first introduce some basic concepts about graph isomorphism, random walk, and quantum walk, separately. Then the related isomorphism algorithms based on quantum walks are discussed in detail.

### 2.1. Graph Isomorphism

**Definition** **1.** *(Graph isomorphism) In graph theory, the isomorphism mapping of graph $G_{1}=\left(V_{1},E_{1}\right)$ and $G_{2}=\left(V_{2},E_{2}\right)$ is a bijective mapping function f between vertex set $V_{1}$ and $V_{2}$:*

(1) $$f:V_{1}\to V_{2},$$

*such that any two vertices $u,v\in V_{1}$ are adjacent if and only if $f\left(u\right)$ adjoins $f\left(v\right)$ in graph $G_{2}$. Whenever an isomorphism mapping exists between the vertex sets of $G_{1}$ and $G_{2}$, these two graphs are called isomorphic and are represented as $G_{1}≅G_{2}$.*

*In this paper, we describe the mapping (f) as **isomorphism mapping** or **isomorphic mapping function**. And because isomorphic mapping (f) consists of many bijective relations, such as $u↔f\left(u\right)$, we use **unit bijection** to represent this kind of bijective relationship and indicate f(u) as a **matching node** or **mapping node** of u.*

In the structural pattern recognition field, graph isomorphism and its generalization, the subgraph isomorphism problem, are considered an important part of pattern matching. Graph isomorphism is also regarded as the exact graph matching problem (graph matching is the process of finding similarities between graphs [15,16]). The subgraph isomorphism is a computational task that determines whether one graph contains a subgraph that is isomorphic to another graph. Some graph isomorphism algorithms can be extended to subgraph isomorphism algorithms, such as the VF-3 algorithm [17].

### 2.2. Random Walk

Random walk on graphs has proved to be a fundamental tool [18]. Given a graph and a starting point in that graph, we randomly choose a neighbor of it, move to this neighbor node, then select a neighbor of this neighbor at random, and move to it. We repeat this process till the termination condition is verified. The random sequence of points choosen in this way is random walk on the graph. Therefore, random walk is a finite Markov chain.

Much of the interest in random walk is stimulated by the important algorithmic applications. Random walk has been used in link prediction in social networks [19] page rank in a search engine [20] and graph classification tasks [21]. Some researchers also apply it to graph representation learning [22] and graph clustering algorithms [23]. Recently, random-walk-based methods have gained more and more attention.

### 2.3. Quantum Walk

The notion of quantum walk was first proposed by physicists in their research [24]. It is the quantum analogue of the classical random walk. Through utilizing quantum superposition state to represent the current walker, quantum walk can perform walking processes with the help of quantum evolution. Therefore, it has some interesting properties not exhibited in the classical random walk. For example, because the quantum evolution is unitary and, hence, reversible, the results of quantum walks do not have a limiting distribution.

As an important model in quantum computation, quantum walk has obvious advantages in many applications, such as fast search algorithms [25] and simulated annealing methods [26]. Besides, it can be used as a toolbox to propose more powerful algorithms in the field of mathematics, including element distinctness problems [27] and graph theory [28].

Generally speaking, quantum walk can be divided into two models, named CTQW [29] and DTQW [30]. Given a graph $G=\left(V,E\right)$, where *V* and *E* indicates the vertex set and edge set, respectively. For CTQW, each vertex *u* in this graph is represented by a quantum basic state $|u\rangle$. And the continuous unitary transformation is utilized to control the state evolution. At time *t*, the state of CTQW on graph *G* is a superposition of all basis states, which can be written as follows:

(2) $$|\varphi_{t}\rangle =\sum_{u\in V}\left(\alpha_{u}\left(t\right)|u\rangle \right),\;\sum_{u\in V}{\left|\alpha_{u}\left(t\right)\right|}^{2}=1,$$

where $\alpha_{u}\left(t\right)\in C$ is the amplitude of basis state $|u\rangle$ in time *t*. When the initial state vector $|\varphi_{0}\rangle$ is given, we can deduce the state vector at any moment from Equation (3). Usually we choose the Laplacian matrix or the adjacency matrix as the parameter matrix *L* in this equation.

(3) $$|\varphi_{t}\rangle =e^{-iLt}|\varphi_{0}\rangle .$$

For DTQW, we represent every edge in the graph by a quantum basic state. This is one of the biggest differences from CTQW. And all basic states are stored in the quantum superposition as follows:

(4) $$|\phi_{t}\rangle =\sum_{\left(u,v\right)\in E}\alpha_{uv}\left(t\right)|uv\rangle ,\sum_{\left(u,v\right)\in E}\alpha_{uv}^{2}\left(t\right)=1,$$

where $|uv\rangle$ denotes the quantum state from vertex *u* to *v* with quantum amplitude $\alpha_{uv}$. Using the Grover diffusion matrices [31], each basic state is transferred as Equation (5).

(5) uv→2dv−1vu+2dv∑∀k∈V,k≠u&(k,v)∈Evk,

where $d\left(v\right)$ represents the degree of vertex *v*. Therefore, the matrix, *U*, which governs the evolution of the walk has entries:

(6) $$\begin{array}{cc} |\phi_{t}\rangle & =U|\phi_{t-1}\rangle \end{array}$$

(7) $$\begin{array}{cc} U_{\left(u,v\right),\left(w,x\right)} & =\left\{\begin{array}{cc} \frac{2}{d\left(x\right)}-\delta_{vw} & if\;u=x\\ 0 & otherwise \end{array},\right. \end{array}$$

for all (u,v),(w,x)∈E, in which $\delta_{ux}$ indicates Kronecker delta. The quantum amplitude for transition (u,v)→(w,x) is given in the $U_{\left(u,v\right),\left(w,x\right)}$ entry of this matrix.

### 2.4. Graph Isomorphism Algorithms Based on Quantum Walk

Because the adjacency matrix of graph is an important parameter in quantum walk, the result of quantum walk is extremely sensitive to the topological structure. And the probability amplitude in evolutionary process could reveal the topological characteristic. Hence, it can be applied in the isomorphism algorithm to improve the performance.

In 2008, Douglas and Wang introduced the Douglas method [32], based on DTQW. After performing quantum walk on two graphs, *G* and *H*, respectively, the probability amplitude of each vertex is obtained, and the two sets of probability amplitudes from two graphs are compared. Vertices with the same probability amplitude are considered as a pair of matching nodes. After analysis, this method can perform isomorphism detection with a complexity of $O(|V{|}^{7})$ and can distinguish most non-isomorphism graphs in experiments.

Likewise, David Emms proposed two graph isomorphism algorithms (Emms-D [1] and Emms-C [12]) based on DTQW and CTQW, respectively. These two methods are both polynomial-time and have the similar mechanisms. Firstly, an auxiliary graph is constructed to connect all vertex pairs with auxiliary nodes. Then, the quantum walks are simulated on this auxiliary graph, respectively. Because the quantum interference on auxiliary nodes or edges could highlight matching nodes, this kind of algorithm can establish vertex bijections based on the comparison of quantum amplitudes.

Inspired by Douglas’ method, Qiang designed an algorithm named Qiang1, which can provide a detailed isomorphism mapping [13] with lower complexity. By adding self-loop to reveal local topological information, another optimized method named Qiang2 was developed [13]. However, these methods have limited performance for regular graph. Recently, we adopted the idea of vertex marking in this algorithm to reduce the impact of symmetry on a regular graph and designed a new algorithm named IsoMarking [14]. Consequently, it performs better compared with Qiang’s methods. However, marking process for each node needs to add an additional node. Such a strategy makes the algorithm become very complex and have low efficiency, especially in large number of nodes. Besides, there are only four marking types in this algorithm. Since the number of vertex marking types is the performance dependent, these four marking types do not adequately reduce the symmetry of the regular graph. Therefore, the performance of this algorithm has a lot of room for improvement.

Despite the lack of rigorous proof in theory, the above methods all show nice performance on ordinary graphs. However, as for the graph with inherent symmetry, such as regular graphs, they cannot achieve a satisfied accuracy in detection of graph isomorphism mapping. Therefore, a novel graph isomorphism algorithm is needed to solve this problem.

## 3. MapEff Algorithm

Based on the analysis of how graphical symmetry affects isomorphism detection, we proposed the MapEff algorithm with its main implementation procedures in this section. To help understand how MapEff obtains the correct isomorphism mapping between regular graphs, a pair of graphs are used as the instance for the description. After completing this algorithm, further discussion about the computation complexity is also presented here.

### 3.1. The Impact of Symmetry in Isomorphism Detection

Regular graphs has strong symmetry, as each vertex has the same number of neighbors. This symmetry introduces many equivalent nodes, and even results in automorphism, which means the graph can map itself through a structure-preserving permutation mapping. Even though quantum walk, as a novel quantum computation model, is sensitive to the topological structure and can establish the unit bijection effectively based on the analysis of probability amplitude, too many candidate unit bijections in a symmetric graph will bring in complex combinations and make it difficult to integrate the correct isomorphic mapping. Therefore, a strong symmetric graph limits the application of these quantum-inspired isomorphism.

**Figure 1:**
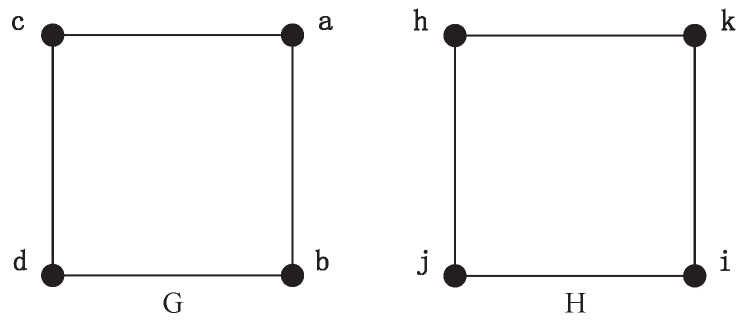
Two regular graphs.

**Figure 2:**
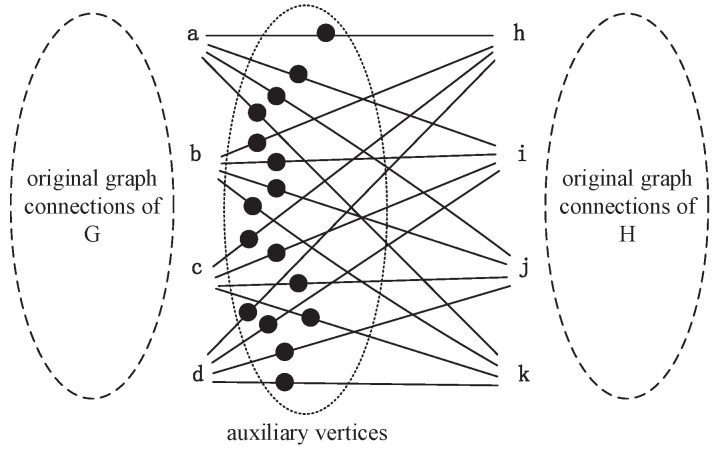
The auxiliary graph by adding auxiliary vertices to connect graph G and H.

For example, two square graphs, *G* and *H*, are shown in Figure 1. Each of them has four equivalent nodes and four edges. Obviously, these two graphs are isomorphic. When we perform the Emms-D algorithm to explore the isomorphic mapping of them, an auxiliary graph is constructed firstly by adding a layer of auxiliary vertices and some extra edges to connect two graphs, as shown in Figure 2. This auxiliary graph has 24 vertices and 40 edges. Each node in the *G* graph is connected to every vertice in *H* by an auxiliary node. We describe the auxiliary node to connect vertex *a* and *h* as $v_{ah}$, and the rest of the auxiliary nodes are expressed similarly. Then the DTQW is simulated on the auxiliary graph. The auxiliary edges with the same probability amplitude reveal the unit bijections in isomorphic mapping. If the auxiliary edges connected to the same auxiliary node have the same probability amplitude, a unit bijection between the corresponding vertices is established. Because both *G* and *H* have four equivalent nodes, we can see that all probability amplitude of auxiliary edges are always the same, as shown in Table 1. That is to say each node in *G* can establish a unit bijection with any node in graph *H*. In order to integrate the unit bijections into isomorphic mapping, we need to choose four unit bijections from 16 candidates. It is not easy to guarantee the combination of these unit bijections can generate the right isomorphic mapping, even under the constrain that each node can only be used once. For instance, the output can be $\left\{a\to h,b\to j,c\to i,d\to k\right\}$. Obviously, this result is not structure-preserving because vertex *b* and *d* are adjacent, but *j* and *k* are not. Therefore, the key issue is to figure out the right one from the candidate unit bijections. Unfortunately, few previous studies have focused on this problem, since most of them just establish unit bijection one by one and simply integrate unit bijections into the isomorphism mapping function. By the random selection of candidate unit bijections, the probability to get the right isomorphic mapping seems unsatisfactory, especially when the graph have too many equivalent vertices. Therefore, an efficient algorithm to map with regular graph isomorphism is necessary.

Unfortunately, most algorithms only make little effort to deal with this problem. They establish unit bijection one by one and simply integrate unit bijections into the isomorphism mapping function. Hence, it is difficult for them to discover the isomorphism mapping between regular graphs. Although the correct graph isomorphic mapping sometimes can be obtained by random selection, the possibility is also very small, especially when the graph have too many equivalent vertices. Therefore, an efficient algorithm for coping with regular graph isomorphism is needed.

### 3.2. Detailed Mechanism of MapEff

In this subsection, we introduce the detailed procedures of MapEff algorithm on two graphs $G=\left\{V_{G},E_{G}\right\}$ and $H=\left\{V_{H},E_{H}\right\}$. The process of this algorithm can be roughly divided into the following steps. First, all candidate matching nodes for each vertex $u_{i}\in G$ are selected based on the NodeMap testing. This strategy is inspired by Emms-D algorithm. It first builds the auxiliary graph through connecting the two testing nodes. Then we utilize the amplitudes computed in DTQW simulation to validate the matching. If there is only one candidate, $v_{j}$, the unit bijection $u_{i}↔v_{j}$ is added to the isomorphic mapping function. For the case of multiple candidates, MapEff needs to identify the most suitable one based on the existing unit bijections. In the most extreme case, if none of the candidates is available, we can conclude that *G* and *H* are not isomorphic. After the unit bijections are established one by one, we can acquire the correct isomorphic mapping function.

To ensure that the DTQW can be simulated, we add a self-loop to each isolated vertex from *G* and *H* which can avoid the case that the degree of vertex is 0. Obviously, this pretreatment does not affect mapping function when two graphs are isomorphic.

**Step 1:** Select candidate matching nodes for each vertex in *G*. To judge whether the node $v_{j}\in H$ is one of candidate mapping nodes of $u_{i}\in G$, an auxiliary graph $GH_{ij}=\left\{V_{ij},E_{ij}\right\}$ defined in Equation (8) is constructed through adding an extra node $c_{ij}$ to connect vertex $u_{i}$ and $v_{j}$.

(8) $$\left\{\begin{array}{c} V_{ij}=V_{G}\cup V_{H}\cup \left\{c_{ij}\right\},\\ E_{ij}=E_{G}\cup E_{H}\cup \left\{\left(u_{i},c_{ij}\right),\left(v_{j},c_{ij}\right)\right\}. \end{array}\right.$$

Then the DTQW is executed on this auxiliary graph. If the quantum amplitudes of basic state representing edge $\left(u_{i},c_{ij}\right)$ and $\left(v_{j},c_{ij}\right)$ are equal, $v_{j}$ is considered as one of candidate mapping nodes of $v_{i}$. This method is inspired by the Emms-D algorithm, which conducts DTQW on a big auxiliary graph that connects all pairs of vertices from two graphs. The difference is that we simplify the construction of auxiliary graph. The details of node matching algorithm are indicated in Algorithm 1.

**Algorithm 1** NodeMap
**Input:**
$G=\left\{V_{G},E_{G}\right\},H=\left\{V_{H},E_{H}\right\},u\in V_{G},v\in V_{H}$

**Output:**
Matchans

1:Construct auxiliary graph through adding an extra node *c* connecting *u* and *v*2:Get the amplitude of basic states representing edge (u,c) and (v,c) based on the DTQW and store them in $\alpha_{uc}$ and $\alpha_{vc}$, respectively.3:
**if** $\alpha_{uc}==\alpha_{vc}$ **then**
4:      Matchans=15:
**else**
6:      Matchans=07:
**end if**



**Step 2:** Preliminary match. If there is only one candidate matching node $v_{j}\in H$ for $u_{i}\in G$, $u_{i}↔v_{j}$ is considered as a unit bijection and added to isomorphic mapping function. When each vertex has multiple candidate matching nodes, we need to accept the first pair of candidate matching nodes as a unit bijection and put it in isomorphic mapping in order to continue.

**Step 3:** Determine the isomorphic mapping. After Step 2, at least one unit bijection in the isomorphic mapping function has been obtained. Next, we need to filter the remaining pairs of matching nodes through multiple iterations. When there are multiple candidate matching nodes for a vertex, we can rely on the existing determined unit bijection to choose the suitable one.

For instance, if we know that $u_{i}↔v_{j}\left(u_{i}\in G,v_{j}\in H\right)$ is a unit bijection and $k_{h}\in H$ is one of the candidate matching node for $k_{g}\in G$, graph $G^{\prime }$ and $H^{\prime }$ defined in Equations (9)–(10) are constructed through adding an edge to connect $k_{g}$ and $u_{i}$, $k_{h}$ and $v_{j}$, respectively. Of course, if $k_{h}$ appears in the previously verified unit bijections, it is not a real candidate matching node and should be ignored.

(9) $$G^{\prime }=\left\{V_{G},E_{G}\cup \left(u_{i},k_{g}\right)\right\}$$

(10) $$H^{\prime }=\left\{V_{H},E_{H}\cup \left(v_{i},k_{h}\right)\right\}$$

Then the node matching algorithm (NodeMap) is performed to determine whether $k_{g}\in G^{\prime }$ and $k_{h}\in H^{\prime }$ are a pair of matching nodes. If $NodeMap\left(G^{\prime },H^{\prime },k_{g},k_{h}\right)==1$, $k_{h}$ is considered as one suitable candidate and the unit bijection $k_{g}↔k_{h}$ is accepted in isomorphic mapping function. Otherwise, this candidate unit bijection is rejected. Through adding auxiliary edges, the symmetry in the original graph can be reduced and MapEff can distinguish the equivalent vertices in a graph. Because the unit bijection $u_{i}↔v_{j}$ is accepted and both graphs $G^{\prime }$ and $H^{\prime }$ use the same constructing method, the matching result of vertices $k_{g}$ and $k_{h}$ is not changed in these updated graphs. That is to say the isomorphic mapping between updated graphs $G^{\prime }$ and $H^{\prime }$ can be effective in graph *G* and *H* when they are isomorphic.

In this way, Algorithm 2 can further screen other candidate matching nodes based on this updated graphs. When unit bijection $k_{g}↔k_{h}$ is accepted, the auxiliary graphs $G^{\prime }$ and $H^{\prime }$ constructed during this iteration is remained for the next round. This means every time a unit bijection is accepted, one edge is added to change the topological structure of the graph, thus reducing the symmetry in the regular graph. After several iterations, we can finally get the right isomorphic mapping function of two isomorphic graphs.

**Algorithm 2** MapEff
**Input:**
$G=\left\{V_{G},E_{G}\right\},H=\left\{V_{H},E_{H}\right\}$
**Output:** isomorphism mapping function Map
  1:Add a loop to each isolated point in the graph *G* and *H*  2:**for** each vertex u∈G **do**  3:      **for** each vertex v∈H **do**  4:           **if** $NodeMap\left(G,H,u,v\right)==1$ **then**  5:                $Candidate\left(u\right)=Candidate\left(u\right)\cup \left\{v\right\}$  6:           **end if**  7:      **end for**  8:
**end for**
  9:**for** each vertex u∈G **do**10:      **if** $\left|Candidate\left(u\right)\right|==1$ **then**11:           $Map=Map\cup \left\{u↔Candidate\left(u\right)\right\}$12:      **end if**13:      **if** $\left|Candidate\left(u\right)\right|==0$ **then**14:           exit(0) (The two graphs are not isomorphism.)15:      **end if**16:
**end for**
17:
**if** Map==∅ **then**
18:      The first appeared candidate unit bijection is added to isomorphism mapping.19:
**end if**
20:**for** each vertex u∈G **do**21:      **for** each $p\in Candidate\left(u\right)$ **do**22:           Construct the auxiliary graph $G^{\prime }$ and $H^{\prime }$23:           **if** $NodeMap\left(G^{\prime },H^{\prime },u,p\right)==1$ **then**24:                $Map=Map\cup \left\{u↔p\right\}$25:                Store the auxiliary graph $G^{\prime }$ and $H^{\prime }$ for next iteration.26:                break27:           **end if**28:      **end for**29:
**end for**



### 3.3. Case Study

This section introduces an example of graph isomorphism detection based on the MapEff algorithm. We perform this algorithm on a pair of square graphs, as shown in Figure 1. Square graph is a kind of regular graph with high symmetry, and we can indicate how the MapEff works out a correct isomorphic mapping by selecting suitable unit bijections on these graph.

**Figure 3:**
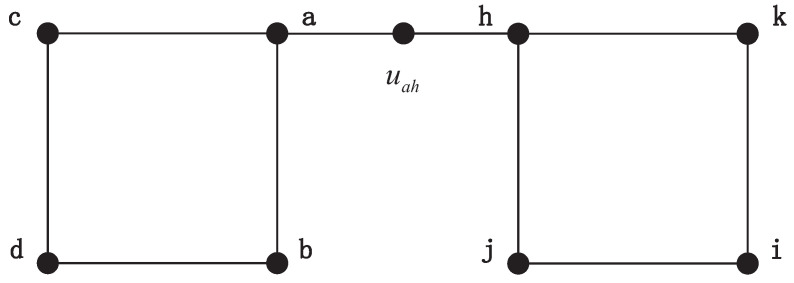
The auxiliary graph by connecting vertex *a* and *h*.

At first, MapEff chooses all the candidate unit bijections based on the DTQW. In each selection, the MapEff constructs an auxiliary graph by connecting two graphs. We assume that it chooses vertex *a* and *h* to determine whether they can form a candidate unit bijection. The graph in Figure 3 is the auxiliary graph in this procedure. Since the auxiliary edges of $\left(a,u_{ah}\right)$ and $\left(h,u_{ah}\right)$ have the same amplitude after simulating DTQW, we can establish a candidate unit bijection a↔h. Through comparing every node pair by this way, all the candidate unit bijections from two graphs are obtained as shown in Table 2. Under the constrain that each vertex is utilized only once, we need to choose four unit bijections and combine them into an isomorphic mapping.

**Figure 4:**
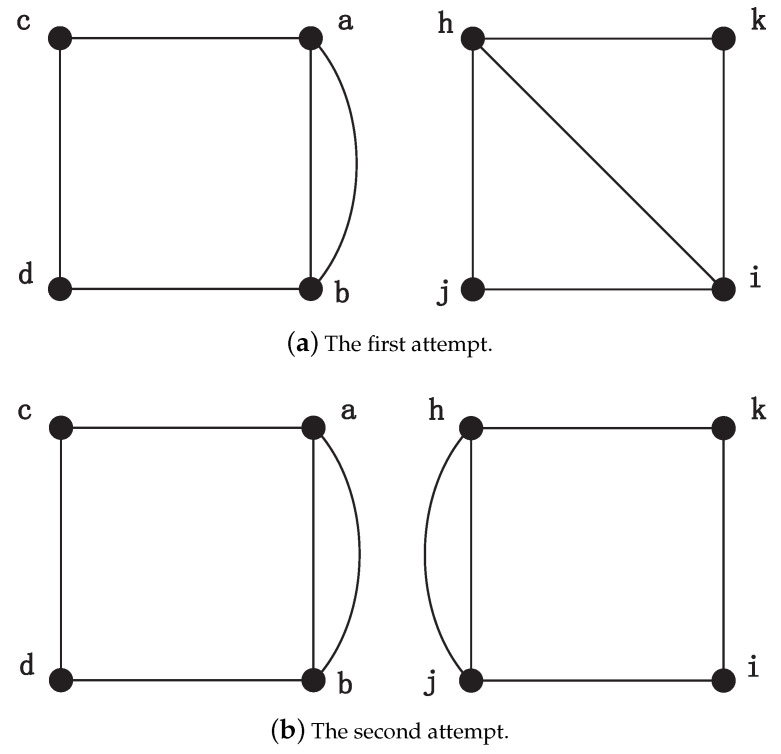
The process of choosing the correct matching vertex of node *b*.

After executing the above procedures, each vertex has four candidate matching nodes. In order to perform the next steps, MapEff selects the first candidate unit bijection a↔h and adds it into the isomorphic mapping. Based on this accepted unit bijection, MapEff decides the matching node of vertex *b*. As the node *h* has appeared in the previously determined unit bijection (a↔h), it will no longer be a matching node for *b*, and the candidate unit bijection (b↔h) will be disregarded directly. Next, to judge whether candidate unit bijection b↔i could be accepted in the mapping isomorphism, it adds an auxiliary edge connecting *a* and *b*, *h* and *i*, respectively, as shown in Figure 4a. Obviously, we can see they are not isomorphic and the NodeMap algorithm also validates this. Therefore, unit bijection b↔i is rejected. Then we try to consider the next candidate unit bijection b↔j. After adding the auxiliary edges to connect vertex (a,b) and (h,j), MapEff constructs two isomorphic new graphs, as shown in Figure 4b. NodeMap proves that vertex *b* and *j* can establish a unit bijection b↔j in the new graphs, so we accept it in the isomorphic mapping function and store the new graphs constructed in this iteration. So far, there are two unit bijections, a↔h and b↔j, in the isomorphism mapping function. Next, MapEff determines the mapping node of vertex *c*.

**Figure 5:**
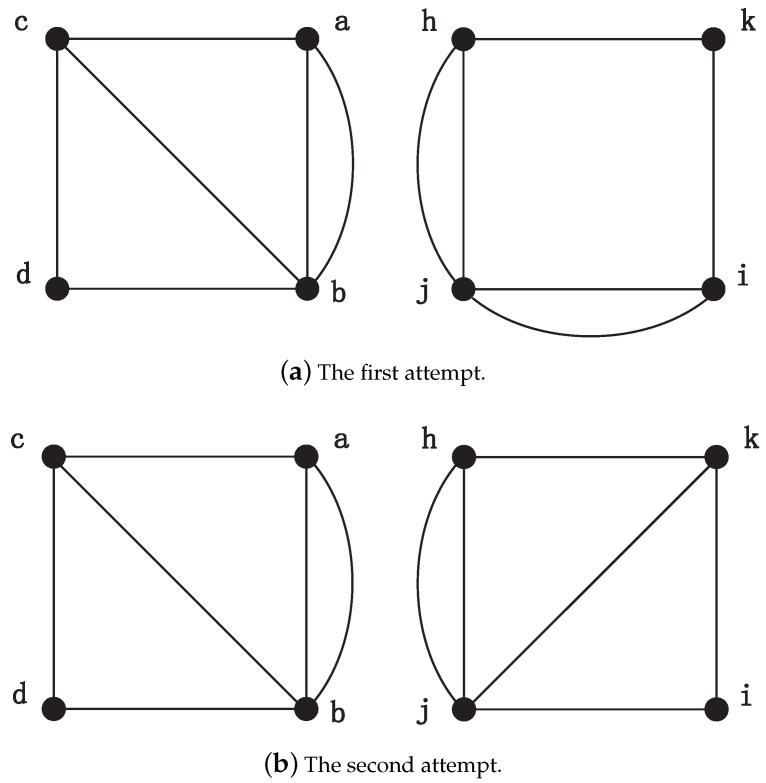
The process of choosing the correct matching node for vertex *c*.

As for the matching vertex of node *c*, nodes *h* and *j* appearing in the previously determined unit bijections (a↔h, b↔j) are directly excluded from the candidates. Then, we choose vertex *j* on the first attempt. Two new graphs are generated by adding auxiliary edges (b,c) and (j,i), respectively, as shown in Figure 5a. Since *c* and *i* are not matched in the new graphs by NodeMap, and node *i* is rejected. When the candidate unit bijection c↔k is considered, MapEff continues to add two auxiliary edges (c,b) and (k,j), respectively. Hence, the new graphs are generated as shown in Figure 5b. This time, these two graphs are isomorphic and the vertex *c* can be mapped to *k* in the NodeMap algorithm. As a result, the unit bijection c↔k is accepted in the isomorphic mapping, and the new generated graph is maintained in the next iteration.

Finally, MapEff has to determine the mapping node of vertex *d* from its four candidates h,i,j,k with the help of verified unit bijection c↔j. Because nodes *h*, *j*, and *k* conflict with a verified unit bijection, they are rejected. Therefore, there is only one node left to verify. When testing the candidate unit bijection d↔i, the vertex *i* can be a mapping node of *d* after performing the NodeMapp algorithm in the new graphs. Hence, MapEff accepts this unit bijection into the isomorphic mapping and outputs the final result $\left\{a↔h,b↔j,c↔k,d↔i\right\}$.

**Figure 6:**
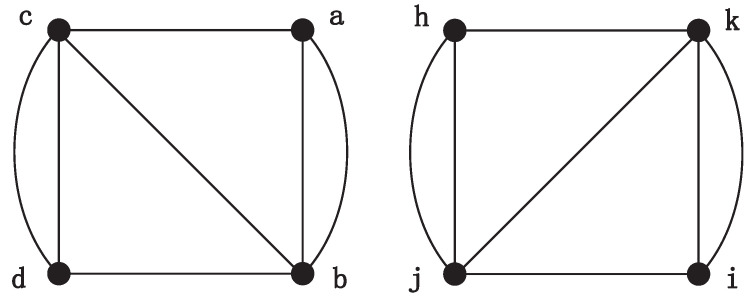
The process of choosing the correct matching node for vertex *d*.

Consequently, in this example, we can see that the unit bijection added into the isomorphic mapping one after another do not conflict with each other. The key point is equivalent nodes are distinguished by adding auxiliary edges. And the newly generated graphs, such as the graphs shown in Figure 4, Figure 5 and Figure 6, also reduce the original symmetry. Although the square graph is highly symmetric and each node has the same number of neighbors, the addition of auxiliary edge is sufficient to distinguish the equivalent vertices. After multiple filters of candidate unit bijections, MapEff can discover a correct isomorphic mapping.

### 3.4. Algorithm Analysis

In this subsection, we discuss the computation complexity of our algorithm on two graphs, *G* and *H*, with the same number of vertices *N*.

According to the discussion in [32], the computational complexity of simulating DTQW on the classical computer is $O\left(N^{4}\right)$ for the graph with *N* nodes. Because the auxiliary graph constructed in Step 1 has 2N+1 points, each simulation of DTQW in Step 1 costs $O\left(4N^{4}\right)$. In this step, this kind of operation is performed $N^{2}$ times. Therefore, its computation complexity is $O\left(N^{6}\right)$.

Next, we need to count the candidate matching nodes for each vertex in *G*. In the worst case, every vertex has *N* candidate matching nodes. So the complexity of Step 2 is $O(N^{2})$.

When selecting the best candidate for each node in Step 3, we need to execute $N^{2}$ DTQW at most. Although the quantum walk we performed in this step is simulated on the auxiliary graph after adding extra edges, the computational complexity of each DTQW is still $O(N^{4})$ because the number of added edges is not greater than *N*. Therefore, the complexity of this step is $O\left(N^{6}\right)$.

After the above analysis, we can conclude that the computational complexity of this algorithm is $O\left(N^{6}+N^{2}+N^{6}\right)=O\left(N^{6}\right)$.

## 4. Experiments

In this section, the experiments on several graph datasets are conducted to evaluate MapEff algorithm. Firstly, we introduce some information about experiment setup. Then the comparison of MapEff with other state-of-art algorithms is presented.

### 4.1. Experimental Setup

We planned to choose some state-of-art isomorphism algorithms to complete the performance comparison with our method. Because the MapEff is a kind of approximation algorithm, we compared it with another algorithm of the same kind.

Although some influential isomorphism algorithms, such as VF2 [8], VF3 [9], LAD [10], RI [2], and Nauty algorithm [11], are optimal and can output perfect results in graph isomorphism detection, their computational complexity is not polynomial-time. In the worst case, the complexity is even exponential or quasi-polynomial. However, MapEff based on quantum walk is similar to the approximation algorithm. The complexity of our algorithm is polynomial-time. It is theoretically superior in computational complexity compared with these optimal algorithms, although it may not always provide an exact result. Because the accuracy of the optimal algorithms is always 100% but they could be less efficient, it is unfair to compare with them no matter in terms of complexity or the accuracy.

We finally chose five state-of-art algorithms (Qiang1, Qiang2, Emms-D, Emms-C, and IsoMarking) for comprehensive comparison because they are polynomial-time and outstanding in isomorphic mapping. Among them, Qiang1 and Emms-C methods are based on the CTQW, while Emms-D is the discrete-time version (i.e., DTQW) algorithm proposed by Emms. And Qiang2 and IsoMarking are declared to perform well on regular graphs.

In our experiments, 16 groups of graphs with 1585 pairs isomorphic graphs were used in total, and the datasets are the same ones referenced in [14]. We concentrated on the accuracy performance of discovering isomorphism mapping since accuracy is the key indicator of approximation algorithm. All·the code implemented in these experiments was programmed in MatlabR2013a and performed on a laptop with an Intel Core-i5 CPU at 2.30 GHz and 8 GB of main memory. Some source codes about the baselines are available from the thesis [13]. And we also implemented the other codes, including Emms-D, Emms-C, and IsoMarking, according to related work [1,12,14].

Two categories of experimental data were involved in our experiment: one set are six groups of ordinary graphs, namely Groups 1–6, while the other are ten groups of regular graphs named Groups 7–16, and identical experimental methods were executed on these two datasets. For each group, we performed more than five algorithms, as well as our MapEff algorithm, on every pair of graph and output the isomorphism mapping result. Then we checked whether the output isomorphic mapping function was structure-preserving based on the definition of graph isomorphism in Definition 1. The verification method was to test all the adjoint nodes and to see whether their mapping nodes were adjacent. This is the same with the verification method in [13,14]. If the result of isomorphism mapping passed the verification, it was deemed to be correct, and vice versa. We calculated the average accuracy in each group for every algorithm and compared the performance of different algorithms.

In this subsection, six groups of ordinary graphs named Groups 1–6 were utilized to conduct the first experiment. Because every group contained 100 pairs of ordinary graphs, there were 600 ordinary graph pairs in total. The detailed information of these groups is introduced in Table 3, where *N* represents the vertex number.

### 4.2. Results on Ordinary Graphs

In Table 4, we show the results for ordinary graphs. Most algorithms performed well in each group. For Group 1 and Groups 3–5, all algorithms carried out perfectly with accuracy of 1.00%. For Group 6, the Qiang1 and Emms-C method had the accuracy of 0.96 and 0.97, respectively, which were only a little worse than other algorithms with accuracy of 1.00. The performance differences among these algorithms are mainly reflected in the Group 3, which has more complicated graphs. The accuracy performance of Qiang1, Qiang2, Emms-C, and IsoMarking algorithms in this group was 64%, 55%, 67% and 99%, respectively. Meanwhile, the Emms-D method results showed the same satisfied accuracy performance as MapeEff, which were 1.00. Compared with other state-of-art algorithms, MapEff and Emms-D perform perfectly for all cases. Their accuracy performance in Groups 1–6 is 100% all the time.

Table 4 shows the results about accuracy for these ordinary graphs. From the table, we can see most algorithms performed well in each group. For Group 1 and Groups 3–5, all algorithms carried out perfectly with an accuracy of 100%, and for Group6, the Qiang1 and Emms-C method had the accuracy of 0.96 and 0.97, which were only a little worse than other algorithms with an accuracy of 1.00. The performance differences between these algorithms are mainly reflected in the Group 3, which had more complicated graphs. Among them, Emms-D and MapeEff show the most satisfactory accuracy of 100%, while the Qiang1, Qiang2, Emms-C, and IsoMarking algorithms are 64%, 55%, 67%, and 99%, respectively. It is apparent from the table that MapEff and Emms-D were outstanding compared with other algorithms, as their accuracy factors in Groups 1–6 are 100% all the time.

Although the performance of other algorithms drops greatly when graphs become more challenging, MapEff and Emms-D could keep a perfect accuracy level. Therefore, we can conclude that MapEff and Emms-D perform the best for the ordinary graph.

### 4.3. Results on Regular Graphs

The second experiment targeted ten groups of regular graphs in Groups 7–16, and there are 985 graph pairs in total. Table 5 indicates the detailed information of each group, and Table 6 represents the accuracy results of six algorithms. Obviously, there are significant differences in the accuracy performance of these algorithms, and among them MapEff stands out in all groups.

As shown in the Table 6, two methods proposed by Emms have the worst performance with the accuracy, usually close to 0. The only exception is the performance of the Emms-C algorithm on Group 15, where the accuracy was 0.5. As for the Qiang1 algorithm, its accuracy is always less than 0.5, except on Group 7. These three methods have poor performance on regular graphs since they hardly consider the detrimental effects of graph symmetry.

Through optimizing regular graphs, Qiang2 and IsoMarking algorithms achieve better performance. Although Qiang2 scores zero in Group 15, it can be considered as an exception from its overall accuracy. So the overall accuracy of Qiang2 is usually about 0.5 to 0.8, much better than Qiang1. And IsoMarking has the accuracy about 0.7 to 0.8, and even achieves a score of 0.92 in Group 7, which is close to that of MapEff. Therefore, we can see that IsoMarking and Qiang2 algorithms have significant improvements in detecting the isomorphism mapping between regular graphs.

As for MapEff, its accuracy is usually higher than 0.9, except for Group 15. In Group 11 and Group 12, the accuracy results even reached 1.00. Even though the worst accuracy among these groups of the MapEff algorithm is 0.875, it is still higher than the best results of Qiang1, Qiang2, Emms-D, and Emms-C algorithms.

There are a few interesting phenomena to note from these experiments. For those regular graph pairs on which MapEff fails to detect the isomorphism mapping, other algorithms also cannot work out the correct results. And there are several regular graphs with strong symmetry where all algorithms cannot deal with them correctly. For these kind of graphs, our algorithm can be further optimized by the help of more verified unit bijections when selecting the matching nodes.

As a result, this experiment indicates that Qiang1, Emms-C, and Emms-D have limited ability to discover the isomorphism mapping between regular graphs. Despite Qiang2 and IsoMarking improving the accuracy, they are still unsatisfactory. Compared with them, MapEff can maintain its good performance. As a result, we can conclude that MapEff performs much better than other state-of-art algorithms on regular graphs.

## 5. Conclusions

In this paper, we proposed an algorithm to discover graph isomorphism mapping named MapEff. It senses the topological information in graphs based on the DTQW. Through adding extra edges to reduce the symmetry in the graph, the equivalent nodes can be distinguished, and the correct unit bijection is determined based on the verified mapping nodes. Therefore, MapEff can keep bijections consistent with each other. In the experiments, MapEff not only achieved a perfect accuracy on ordinary graphs but also significantly outperformed other state-of-art methods for regular graphs with inherent symmetry. In the future, we plan to further optimize our algorithm in order to make it more efficient and effective. Likewise, we want to study the quantum walk mechanism more deeply, so that it can be explored to develop an algorithm that can detect isomorphic mapping in some extremely difficult structures.

## Figures and Tables

**Table 1:** The amplitude of auxiliary edge in discrete-time quantum walk (DTQW) simulation.

Auxiliary Edges	T = 1.0	T = 2.0	T = 3.0
($a,v_{ah}$)	0.1667	−0.0556	0.0741
($a,v_{ai}$)	0.1667	−0.0556	0.0741
($a,v_{aj}$)	0.1667	−0.0556	0.0741
($a,v_{ak}$)	0.1667	−0.0556	0.0741
⋮	⋮	⋮	⋮
($d,v_{dh}$)	0.1667	−0.0556	0.0741
($d,v_{di}$)	0.1667	−0.0556	0.0741
($d,v_{dj}$)	0.1667	−0.0556	0.0741
($d,v_{dk}$)	0.1667	−0.0556	0.0741

**Table 2:** All candidate unit bijections after performing Step 1 of MapEff algorithm.

a↔h	b↔h	c↔h	d↔h
a↔i	b↔i	c↔i	d↔i
a↔j	b↔j	c↔j	d↔j
a↔k	b↔k	c↔k	d↔k

**Table 3:** Information about the graph groups used in first experiments.

Group Name	Graph Number	N	Average Degree
Group 1	100	17	10.24
Group 2	100	34	4.53
Group 3	100	18	3.00
Group 4	100	18	4.11
Group 5	100	20	3.80
Group 6	100	10	3.00

**Table 4:** Accuracy results for ordinary graphs.

Group	Qiang1	Qiang2	Emms-C	Emms-D	IsoMarking	MapEff
Group 1	1.00	1.00	1.00	1.00	1.00	**1.00**
Group 2	0.64	0.55	0.67	1.00	0.99	**1.00**
Group 3	1.00	1.00	1.00	1.00	1.00	**1.00**
Group 4	1.00	1.00	1.00	1.00	1.00	**1.00**
Group 5	1.00	1.00	1.00	1.00	1.00	**1.00**
Group 6	0.96	1.00	0.97	1.00	1.00	**1.00**

**Table 5:** Information about the graph groups used in second experiments.

Group Name	Graph Number	N	Average Degree
Group 7	100	30	3.00
Group 8	149	16	3.00
Group 9	100	14	4.00
Group 10	200	14	3.00
Group 11	100	11	6.00
Group 12	100	11	4.00
Group 13	85	12	3.00
Group 14	60	10	5.00
Group 15	32	20	3.00
Group 16	59	10	4.00

**Table 6:** Accuracy results for regular graphs.

Group	Qiang1	Qiang2	Emms-C	Emms-D	IsoMarking	MapEff
Group 7	0.64	0.64	0	0	0.92	**0.94**
Group 8	0.38	0.68	0	0.03	0.90	**0.99**
Group 9	0.28	0.56	0	0.04	0.68	**0.98**
Group 10	0.28	0.59	0	0.04	0.87	**0.99**
Group 11	0.44	0.83	0	0	0.86	**1.00**
Group 12	0.40	0.83	0.02	0.04	0.90	**1.00**
Group 13	0.15	0.58	0.01	0.09	0.84	**0.99**
Group 14	0.15	0.62	0	0.05	0.85	**0.95**
Group 15	0	0	0.50	0	0.75	**0.88**
Group 16	0.22	0.61	0	0.02	0.75	**0.97**

## Abbreviations

The following abbreviations are used in this manuscript:

CTQW	Continuous-time quantum walk
DTQW	Discrete-time quantum walk
NP	Nondeterministic Polynomial-time
